# Supplementary material for: Splice-Junction-Based Mapping of Alternative Isoforms in the Human Proteome
Source: Cell Rep. Author manuscript; Available in PMC 2020 Jan 15. (PMC6961840; doi:10.1016/j.celrep.2019.11.026)
Supplement: 2 [file NIHMS1546469-supplement-2.zip › DF1/PXD010154/Liver/Liver_70_CPT1B_RPHQEVPDQPFQFTVTPDGVDFR.pdf]

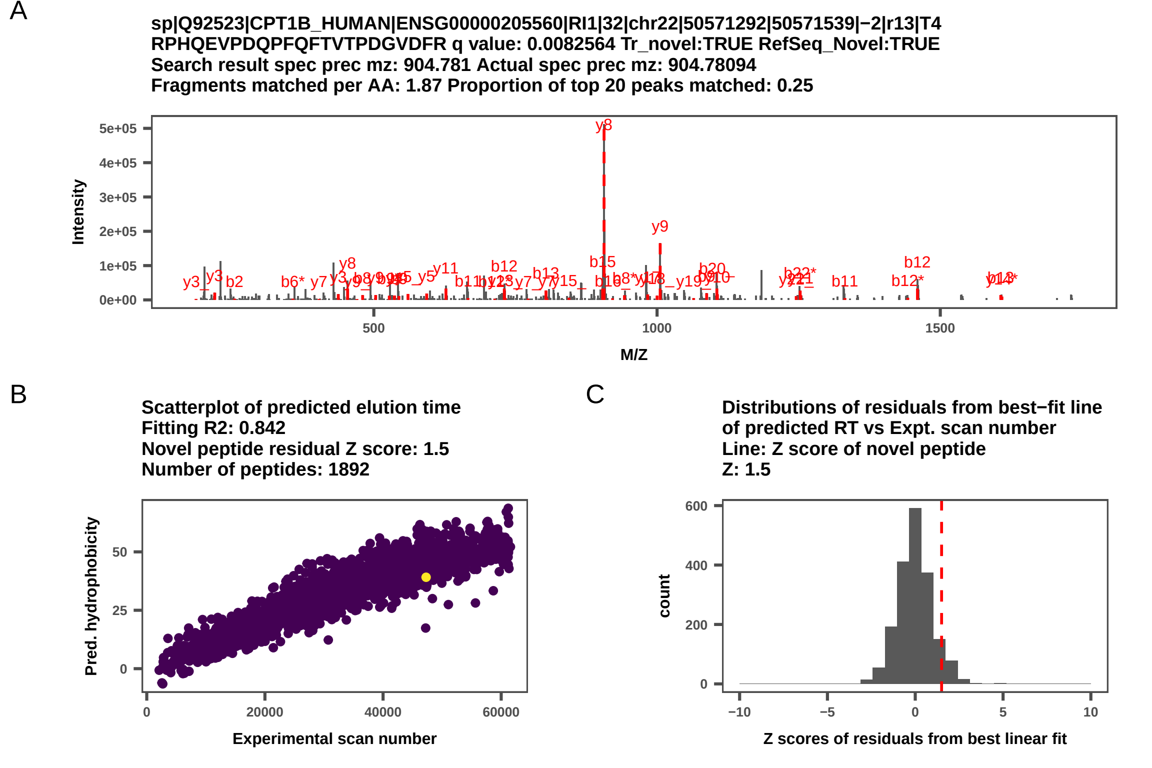
A
sp|Q92523|CPT1B_HUMAN|ENSG00000205560|RI1|32|chr22|50571292|50571539|−2|r13|T4
RPHQEVPDQPFQFTVTPDGVDFR q value: 0.0082564 Tr_novel:TRUE RefSeq_Novel:TRUE
Search result spec prec mz: 904.781 Actual spec prec mz: 904.78094
Fragments matched per AA: 1.87 Proportion of top 20 peaks matched: 0.25
Intensity
5e+05
4e+05
3e+05
2e+05
1e+05
0e+00
y8
y9
b15
b12
b20
b11
b12*
y11
b2
b6*
b13
500
1000
1500
M/Z
B
Scatterplot of predicted elution time
Fitting R2: 0.842
Novel peptide residual Z score: 1.5
Number of peptides: 1892
Pred. hydrophobicity
50
25
0
0
20000
40000
60000
Experimental scan number
C
Distributions of residuals from best−fit line
of predicted RT vs Expt. scan number
Line: Z score of novel peptide
Z: 1.5
count
600
400
200
0
−10
−5
0
5
10
Z scores of residuals from best linear fit
